# Enhancing Anemia Detection With Non-invasive Anemia Detection With AI (NiADA): Insights From Clinical Validations and Physician Observations

**DOI:** 10.7759/cureus.76369

**Published:** 2024-12-25

**Authors:** Vipul Sharma, Debjeet Das, Sagarika Sarkar, Suvraraj Das, Pasang Lahmu Sherpa, Arpan Ray, Farhad Ahamed, Jhuma Nandi, Mou Nandi, Krishanu Banerjee

**Affiliations:** 1 Artificial Intelligence, Monere AI, Delhi, IND; 2 Artificial Intelligence, Monere AI, Kolkata, IND; 3 Pathology, ESI Corporation Medical College and Hospital, Kolkata, IND; 4 Uro-Oncology and Robotics, HCG Cancer Centre, Kolkata, IND; 5 Medicine, North Bengal Medical College and Hospital, Siliguri, IND; 6 Community Medicine and Family Medicine, All India Institute of Medical Sciences, Kalyani, IND; 7 Artificial Intelligence, Monere AI, New Jersey, USA; 8 Artificial Intelligence, Monere AI, Lehi, USA

**Keywords:** anemia, artificial intelligence, hemoglobin monitoring, image processing, non-invasive, smart phone app

## Abstract

Background

Anemia, a critical public health issue, affects nearly two billion people globally. Frequent monitoring of blood hemoglobin levels is essential for managing its burden. While point-of-care testing (POCT) devices facilitate hemoglobin testing in resource-limited settings, most are invasive and have inherent limitations. The Non-Invasive Anemia Detection App (NiADA) (Monere, UT) offers a non-invasive alternative, utilizing artificial intelligence (AI) to estimate hemoglobin levels from images of the lower eyelid. This low-cost, real-time solution employs a custom computer vision deep-learning algorithm for hemoglobin levels, offering significant potential for early diagnosis and management of anemia.

Methods

This study evaluated NiADA in two phases. In the first phase, its performance was compared to laboratory measurements and the minimally invasive POCT device, Hemocue Hb 301. In this study, the current version of NiADA version 2 (V2) is also compared against the previous version of NiADA version 1 (V1) to show the improvement in the last six months. In the second phase, NiADA’s results were compared against hemoglobin estimations made by a group of medical professionals, as well as against lab analyzers. For both phases, NiADA performance was evaluated using the Bland-Altman plot, regression coefficients, percentage of acceptable limit, Pearson correlation coefficient, and Lin’s concordance correlation coefficient.

Results

The mean difference between NiADA-V2 and laboratory-estimated hemoglobin values was -0.11 g/dL, with limits of agreement (LOA) ranging from +2.86 to -2.64 g/dL, where the upper limit is comparable with HemoCue. The NiADA-V2-acceptable range (percentage of samples falling within ±1 g/dL absolute error) increased to 54% compared to 40% in NiADA-V1. Additionally, NiADA outperformed medical professionals, showing a mean difference of 0.07 g/dL compared to medical professionals' 0.42 g/dL.

Conclusion

NiADA, as a non-invasive application, exhibits performance comparable to minimally invasive tools and other POCT devices. Its accuracy exceeds that of medical professionals, making it a viable option for anemia screening and monitoring, particularly in community medicine and regions with limited healthcare resources.

## Introduction

According to the WHO Global Database, anemia affects 1.92 billion people globally, which corresponds to 24.8% of the world's population. The highest prevalence is among preschool-age children, while the lowest is in men. Worldwide, 47.4% of preschool children, 41.8% of pregnant women, and 30.2% of non-pregnant women are anemic [1].

Anemia associated with pregnancy is the leading cause of morbidity and mortality of pregnant women in developing countries affecting both maternal and fetal health outcomes [2,3]. For pregnant women, in over 80% of the countries, anemia poses a moderate to severe public health problem. Globally, anemia is estimated to cause more than 115,000 maternal and 591,000 perinatal deaths annually [4]. Common etiological factors of anemia include nutritional deficiencies, infections, inflammatory diseases, and hemoglobinopathies [5]. Iron deficiency anemia was estimated to account for 22% of maternal deaths in 2019 [6]. The population groups at risk of developing anemia comprise children under five years of age, especially infants and children under two years of age, menstruating adolescent girls, and pregnant and postpartum women [7]. Global estimates state that anemia affects half a billion women between 15 and 49 years of age and 269 million children aged 6-59 months (about five years). In 2019, anemia was estimated to affect 30% (539 million) of non-pregnant women and 37% (32 million) of pregnant women 15-49 years of age [7]. The prevalence of anemia in women 15-49 years of age, based on pregnancy status, is indicator numbered 2.2.3 of the UN Sustainable Development Goals, and the aim of reducing the anemia prevalence by half in women of reproductive age by 2030 is a continuation of the 2025 global nutrition targets endorsed by the World Health Assembly (WHA) [8].

Regular monitoring is indispensable to tackling this grave public health problem, which affects populations in both developed and developing nations. Early detection of anemia using a non-invasive technique promises to go a long way in reducing the burden of anemia prevalence among different age groups worldwide.

The smartphone-based application Non-invasive Anemia Detection App (NiADA) leverages artificial intelligence (AI) technology to detect anemia by analyzing images of the inner eyelid, providing instant hemoglobin estimations. Previous research has explored hemoglobin estimation through eyelid color using custom state-of-the-art (SOTA) algorithms with notable success. For instance, a study by Suner et al. at Brown University utilized smartphone cameras and custom algorithms to predict hemoglobin levels [9]. Similarly, Ghosal et al. outlined comparable approaches achieving reasonable accuracy [10], though these experiments were conducted on smaller sample sizes.

What sets NiADA apart is its reliance on a significantly larger dataset around 50,000 samples and the application of advanced AI techniques to map eyelid images to laboratory-confirmed hemoglobin values. Moreover, NiADA is cost-effective, requiring no additional devices, and is entirely non-invasive.

Since NiADA is based on artificial image processing, its performance can be further enhanced through larger datasets and more sophisticated algorithms. By filtering out low-quality data and undergoing rigorous retraining, NiADA continues to improve. This paper introduces NiADA-V2, an enhanced version of the application, validated using clinical data from February 2024. We compare the performance of NiADA-V2 with clinical observations by doctors and demonstrate its consistency across datasets.

## Materials and methods

NiADA aims to provide a non-invasive, accessible tool for hemoglobin level determination, particularly useful in regions with limited access to medical resources. NiADA uses a machine learning pipeline to identify the lower eyelid portion in the eye image and to determine hemoglobin levels from the lower eyelid portion. The image can be obtained using an ordinary mobile device camera. The pipeline includes additional steps such as adjusting the white balance, de-noising the image, and excluding images with red or yellow values above certain thresholds. NiADA leverages deep learning models, specifically convolutional neural networks (CNNs). To enhance model robustness and generalization, advanced data augmentation techniques such as generative adversarial networks (GANs), MixUp, and stain augmentation were employed.

Clinical validation revisited - study procedure 

NiADA’s first clinical validation was conducted at the All India Institute of Medical Sciences (AIIMS) Kalyani, West Bengal, India, between February and April 2024, in the Outpatient Department (OPD). Samples were collected from patients who provided written informed consent, and the study was approved by the Institutional Ethics Committee, AIIMS Kalyani (approval number: IEC/AIIMS/Kalyani/certificate/2024/04). During this three-month period, hemoglobin results were collected from 607 patients using two NABL-certified laboratories, alongside HemoCue results and NiADA predictions. The results of this study have been previously published by NiADA [11]. While NiADA demonstrated reasonable performance during the initial validation, the model has since been significantly improved through training on increased sample size. NiADA-V2, the enhanced model, was tested using the same set of samples from the AIIMS validation study. The results were reevaluated, demonstrating the impact of the updated training and model enhancements.

Doctor evaluation - study procedure 

In a separate experimental setting, we evaluated NiADA's performance in comparison to medical professionals. Estimating hemoglobin levels from eyelid images has long been recognized in medical science. Our objective was to determine whether NiADA could outperform human expertise in this task.

For the study, 340 randomly selected lower eyelid images of a single eye were provided to 15 doctors from North Bengal Medical College. The participants were either postgraduate students or fully trained pathologists. Poor-quality images - such as those that were blurred, dark, out of focus, or overexposed - were excluded from the study. Images were captured using various smartphones under similar illumination conditions.

The doctors carefully examined the pallor and color contrast of the eyelid images and provided their best estimated hemoglobin levels. Most images were evaluated by multiple doctors, and the average of their estimates was considered for each sample.

Simultaneously, the same set of images was processed by NiADA to predict hemoglobin levels. The predictions made by the doctors and NiADA were then compared against laboratory-measured hemoglobin levels to assess accuracy.

Inclusion and exclusion criteria 

The study included adult females, adult males, pregnant females, and pediatric participants aged 2-18 years. Patients who were severely ill or debilitated, those with eye trauma, scarring over the eye, or any pathological or physiological conditions that hindered photography of the lower palpebral conjunctiva were excluded. Additionally, all individuals with hemoglobinopathies or other conditions that obstructed clear visualization of the lower palpebral conjunctiva were excluded from the study.

Statistical analysis 

Statistical analysis was done using Python language (version 3.10; Python Software Foundation, Wilmington, DE). Continuous variables were reported through mean (SD) and categorical variables through proportions. The mean ± SD of hemoglobin estimates from the NiADA-V2 application, HemoCue Hb 301, and Sysmex autoanalyzer were compared. The mean difference ± SD between NiADA-V2 and the autoanalyzer, along with the limits of agreement, was calculated based on the Bland-Altman plot. Lab value vs. NiADA-V2 value regression results also showed and compared key metrics such as the slope, the intercept of the trend line with R-square, and the Pearson correlation coefficient.

## Results

AIIMS study 

Study Population Statistics 

A total of 607 samples were initially collected; however, 51 were excluded due to poor picture quality, resulting in an 8.1% loss of the total dataset. Hemoglobin results from two NABL-certified laboratories, AIIMS and Lupin, were compared. Only readings that were common to both laboratories and fell within the 95% limits of agreement (LOA) in the Bland-Altman analysis were included in the study.

In the Bland-Altman analysis, the average of the two methods was plotted against the differences in their results. Performance was assessed using the mean difference and the 95% confidence interval, calculated as 1.96 times the standard deviation (SD). The LOA was defined as follows:

Upper LOA = mean + (1.96 × SD) 

Lower LOA = mean - (1.96 × SD)

Samples falling outside these limits were considered outliers and excluded from further analysis. Additionally, samples not present in both laboratories were removed. After these exclusions, the sample size was reduced to 421.

Using the NiADA-V2 feature to filter out poor-quality images, a final total of 336 participants were included in the analysis. The participants' ages ranged from 6-80 years, comprising 170 adult females (50.6%), 139 adult males (41.4%), and 27 pediatric patients (8%). The mean ages were as follows:

Adult females: 44 ± 11 years

Adult males: 48 ± 14 years

Children: 13 ± 4 years

Comparison of NiADA, HemoCue, and lab analyzer 

**Figure 1:**
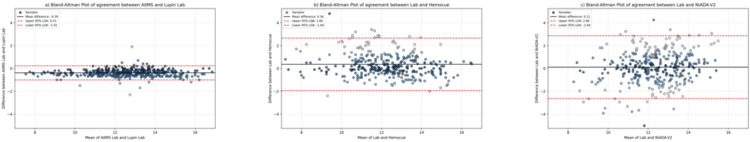
a) Bland-Altman plot of the agreement between AIIMS and Lupin Lab. b) Bland-Altman plot of the agreement between Lupin Lab and HemoCue. c) Bland-Altman plot of the agreement between Lupin Lab and NiADA-V2

NiADA-V2's performance was evaluated against laboratory results and a point-of-care solution, HemoCue, using a Bland-Altman plot. Figure 1 (a) shows the comparison between the two labs, which show good alignment. However, the difference between the upper and lower LOA is 1.24 (ranging from +0.23 to -1.01), with a few outliers. In the later part of the analysis, these outliers were removed, and only AIIMS lab data (referred to as lab) were used. The Bland-Altman plots comparing HemoCue and NiADA-V2 against the AIIMS Lab are shown in Figures 1b, 1c. NiADA-V2 has a lower mean error than HemoCue, but its upper and lower LOAs are wider. Additionally, NiADA-V2 has more outliers than HemoCue. However, HemoCue and NiADA-V2 have shown overall good agreement.

**Figure 2:**
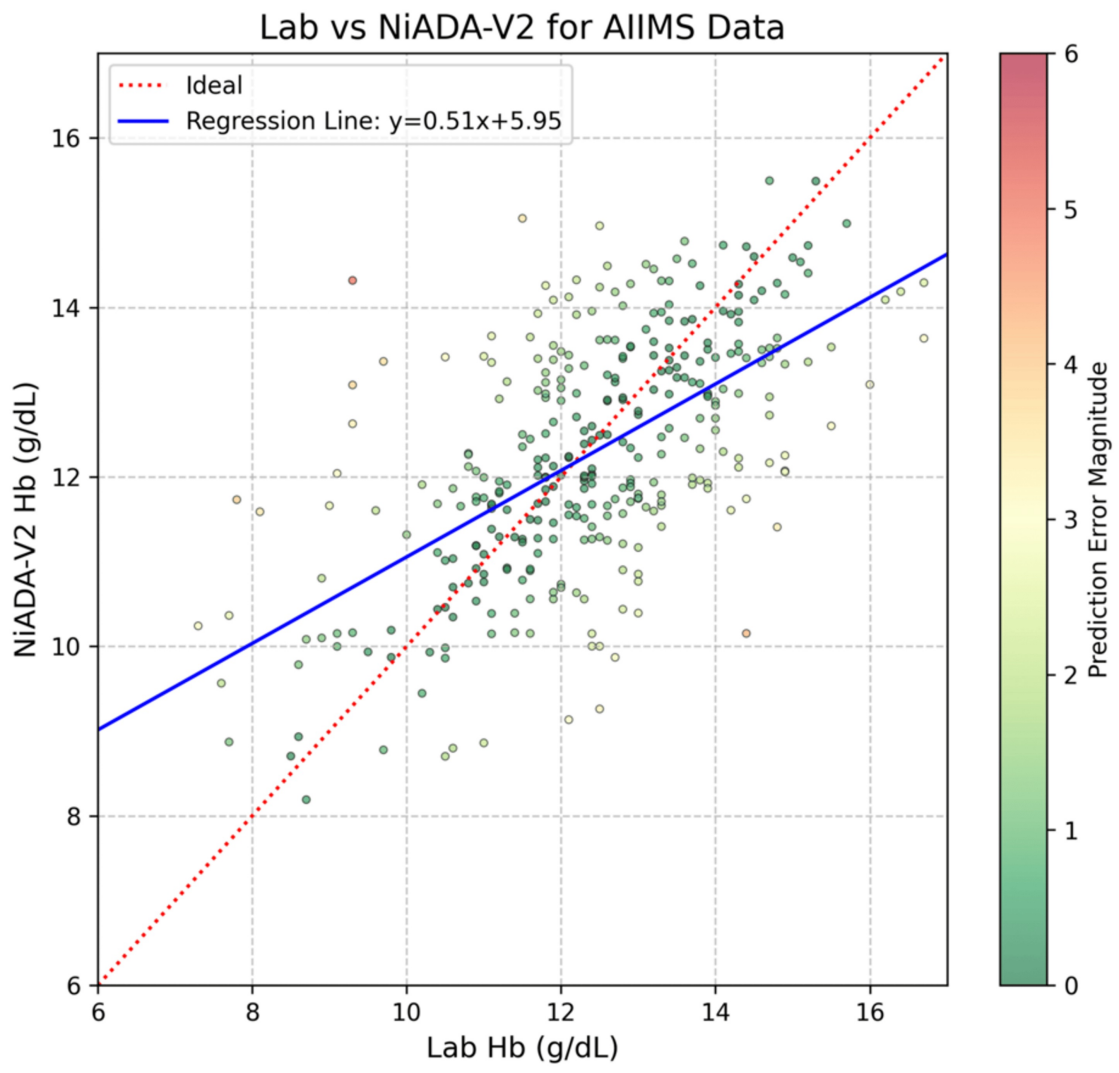
Lab vs NiADA V2 prediction plot with a regression line

Figure 2 illustrates the comparison between laboratory hemoglobin measurements and NiADA-V2 predictions, demonstrating a moderate relationship represented by the regression equation y=0.51x+5.95, where the slope is 0.51 and the intercept 5.95. The trend line aligns well with the data, particularly within the mid-range of hemoglobin values. However, while the model captures the overall trend effectively, opportunities for improvement remain at the lower and higher ends of the hemoglobin spectrum.

**Figure 3:**
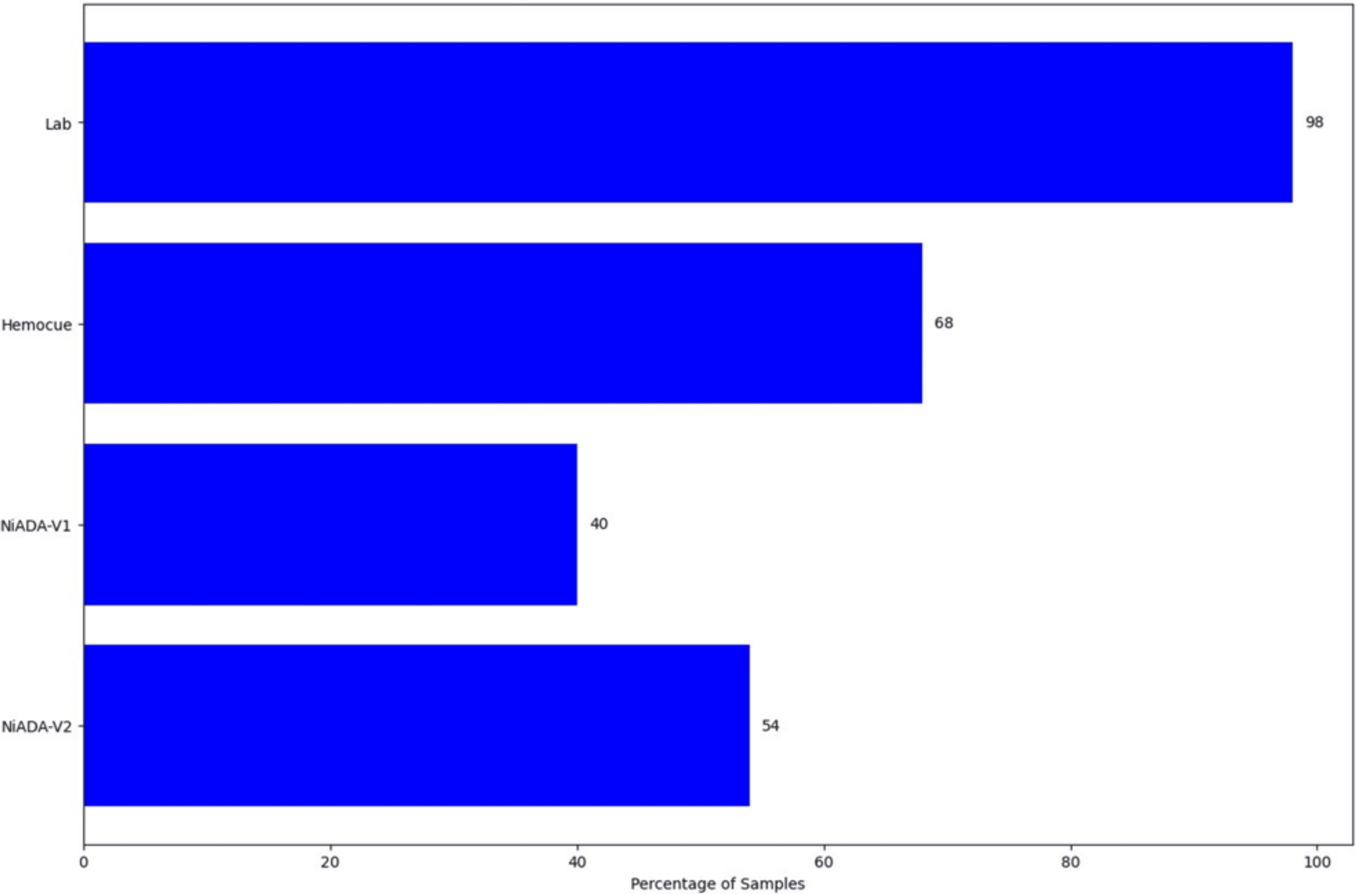
Comparison of absolute error within ±1 for Lupin Lab, HemoCue, NiADA-V1, and NiADA-V2

In hemoglobin measurement, an absolute error of ±1 is considered the acceptable limit. In Figure 3, we compare the percentage of samples with an absolute error of 1. The AIIMS lab serves as the reference (source of truth), and the values from Lupin Lab, HemoCue, NiADA-V1 (here NiADA-V1 refers to the April 2024 version of our model as mentioned [9]), and NiADA-V2 are compared against it. Lupin Lab achieved 98% of samples within the absolute error of 1, while HemoCue had 68%, and NiADA-V2 had 54%. However, NiADA-V2 has improved by approximately 14% over the past six months.

Table 1 summarizes the comparison in detail. In Table 1, We observe an improvement of NiADA-V2 over NiADA-V1. The mean absolute error (MAE) for the range of 6-17 g/dL has decreased from 1.42 g/dL to 1.1 g/dL, and the percentage of samples with an absolute error of 1 or less has increased to 54%. NiADA-V2 has less bias of 0.11 compared to HemoCue, which is 0.36. The number of samples with an absolute error greater than 2.5 has also decreased significantly. The LOA is another measure of the spread of the distribution, which is measured by bias +/- 1.96* standard deviation. The NiADA-V2 upper limit is comparable with HemoCue, but for the lower end, HemoCue is better than NiADA-V2. Moreover, a significant improvement is observed for NiADA-V2 over NiADA-V1 in the R-squared (R²) value. From the table, it can be seen that HemoCue is still better than NiADA-V2, but NiADA made great progress over NiADA-V1.

**Table 1:** Comparison between HemoCue, NiADA-V1, and NiADA-V2 LOA^*^-  upper limit/lower limit  (upper limit = bias + 1.96 * standard deviation  and lower limit = bias - 1.96 * standard deviation)

Method	MAE	Bias	LOA *	% of samples under 1 absolute error	% of samples over 2.5 absolute error	R square
Hemocue	0.82	0.36	+2.66 /-1.94	68	4.5	0.47
NiADA-V1	1.42	0.8	+3.87 /-2.26	40	25	0.07
NiADA-V2	1.1	0.11	+2.86 /-2.64	54	8.3	0.30

Comparison with doctors

Study Population Statistics

The study initially included 340 unique eyelid images. After applying the NiADA-V2 feature to filter out poor-quality images, 309 participants were included in the final analysis. Of these 309 samples, 184 are adult females, 98 are adult males, and 27 are children (age range: 5-18). The age range of the participants spans from 5 to 80 years, with an average age of 42.7 years. Complete blood count (CBC) results are available for all participants, with hemoglobin values ranging from 6.9 to 17.0 g/dL. The average hemoglobin value is 12.06 g/dL, with a standard deviation of 1.74. Among this population, 125 adult females are anemic (Lab CBC Hb ≤ 12 g/dL), 42 adult males are anemic (Lab CBC Hb ≤ 13 g/dL), and four children are anemic (Lab CBC Hb ≤ 11 g/dL). 

Comparison between doctors' estimation and NiADA-V2 

**Figure 4:**
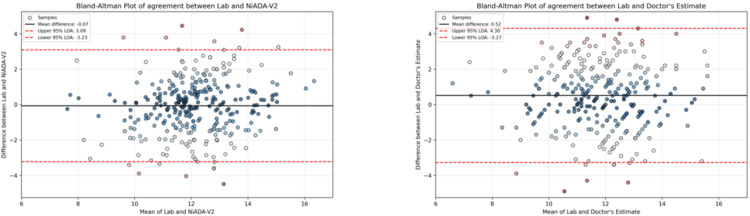
Lab vs NiADA-V2 (left) and Lab vs doctors' estimate (right)

Figure 4 presents the Bland-Altman plot illustrating the agreement between hemoglobin estimates from NiADA-V2 and those provided by doctors. The plot shows that NiADA-V2 has a narrower LOA, ranging from -3.23 to +3.09 g/dL, compared to doctors' estimates, which range from -3.27 to +4.30 g/dL. This narrower range suggests that NiADA-V2 provides more consistent estimates overall, as the variability in its measurements is smaller. However, it is notable that doctors' estimates exhibit good accuracy at lower hemoglobin levels, indicating that their clinical judgment might be more precise in identifying lower hemoglobin values, even though their estimates have a wider range of agreement overall.

**Figure 5:**
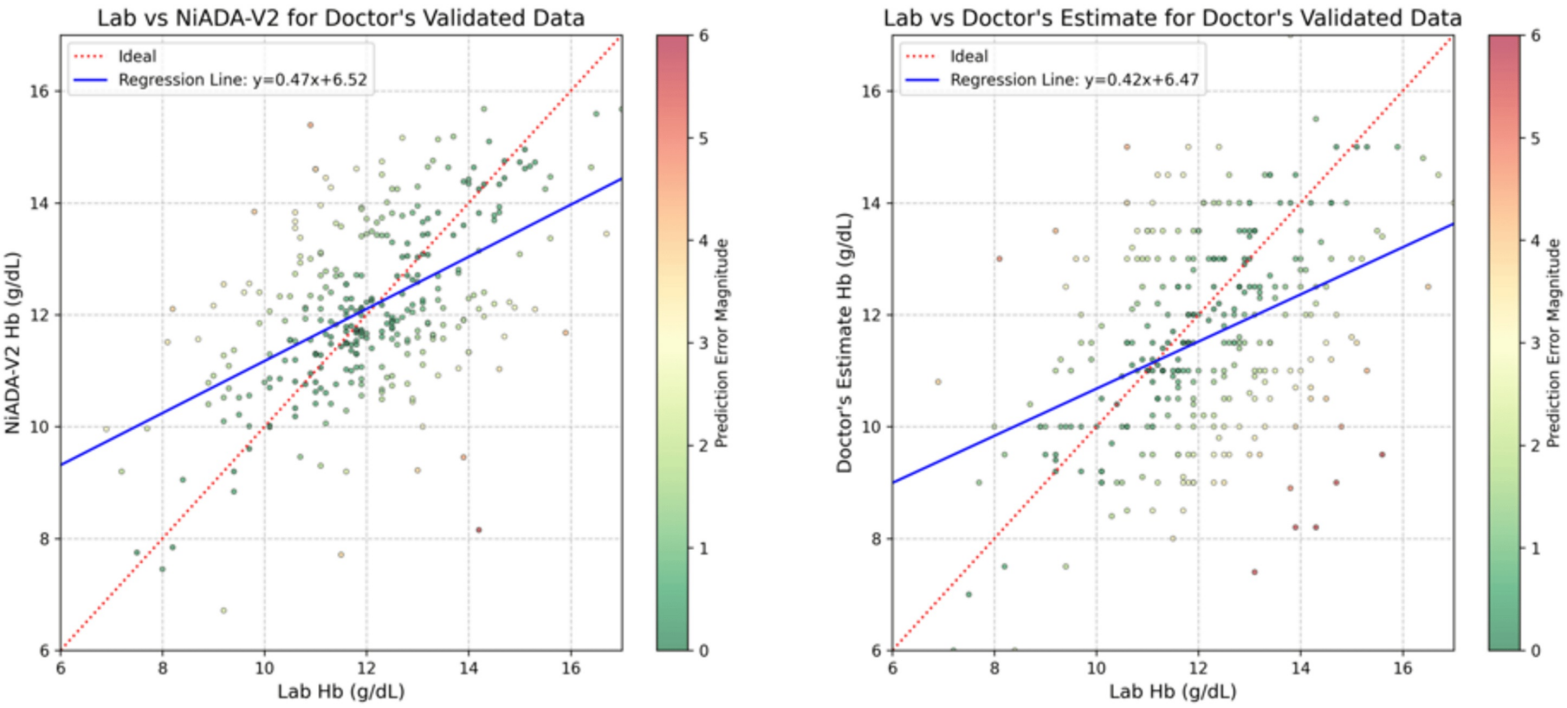
Lab vs NiADA-V2 prediction plot, with regression line (left) and Lab vs doctors' estimate predictions plot, with regression line (right)

Figure 5 highlights the superior performance of NiADA-V2 compared to doctors' hemoglobin estimates. NiADA-V2 demonstrates greater consistency with fewer outliers, indicating more reliable predictions across the dataset. The regression analysis further supports this observation, as the regression line for NiADA-V2 aligns more closely with the actual data points. Additionally, the slope of NiADA-V2's regression line (0.47) is steeper than the slope derived from the average of multiple doctors' estimates (0.42). This steeper slope suggests that NiADA-V2 captures the relationship between the predicted and true hemoglobin values more accurately, reflecting a stronger predictive capability.

Table 2 summarizes the comparison of AIIMS data and doctor validation data in terms of Pearson correlation coefficient (ρ) and Lin's concordance correlation coefficient (ρc). For AIIMS, NiADA-V2 has shown a decent correlation (ρ = 0.6 and ρc = 0.59), though lower than the invasive device HemoCue (ρ = 0.79 and ρc = 0.77). For doctors' validation, NiADA-V2 showed a better correlation than the doctor’s estimates, with a correlation of ρ = 0.52 and ρc = 0.52, compared to doctors' estimates, which had a correlation of ρ = 0.41 and ρc = 0.39. This suggests that NiADA-V2 can be a reliable device for hemoglobin estimation.

**Table 2:** Comparison between Pearson correlation coefficient (ρ) and Lin's concordance correlation coefficient (ρc) for AIIMS and doctors' validated data

Data	Comparison between	Pearson correlation coefficient (ρ)	Lin’s concordance correlation coefficient (ρc)
AIIMS	Lab vs NiADA-V2	0.60	0.59
	Lab vs Hemocue	0.79	0.77
Doctors' Validation	Lab vs NiADA-V2	0.52	0.52
	Lab vs Doctor Estimate	0.41	0.39

## Discussion

The current study evaluated the performance of NiADA, an AI-based smartphone application for hemoglobin estimation, across two distinct scenarios. In the first scenario, NiADA was validated against two NABL-certified laboratories and a point-of-care invasive hemoglobin measuring device, HemoCue. In the second scenario, its performance was compared to that of a group of medical professionals who visually inspected lower eyelid images. A similar study by Hung et al. [12] evaluated the performance of medical professionals in a comparable setting and got similar results. Notably, NiADA outperformed the medical professionals in the current study, suggesting that, in situations where a doctor is unavailable, NiADA could serve as a reliable alternative for hemoglobin estimation.

This finding holds particular significance for regions in Southeast Asia and Africa, where rural areas often lack laboratory facilities. In such settings, primary healthcare providers typically estimate hemoglobin levels through visual inspection of the lower eyelid, a method prone to variability. NiADA offers a more reliable and accessible alternative for hemoglobin assessment in community medicine.

The first study revealed that NiADA shows significant agreement with laboratory estimation and the HemoCue Hb 301 with some exceptions. The low mean bias and limits of agreement make the NiADA application usable in community settings with reasonable confidence. Although HemoCue remains more accurate, particularly at lower hemoglobin levels, NiADA-V2 has shown substantial improvements over its predecessor, NiADA-V1, in the range of 6-17 g/dL. These advancements were achieved within six months, underscoring the scalability and adaptability of the AI technology underlying NiADA. Such progress highlights the potential of AI-driven solutions to bridge critical healthcare gaps in resource-limited settings.

NiADA demonstrates performance comparable to, and in some cases superior to, other non-invasive hemoglobin measuring devices. For instance, AnemoCheck (Sanguina, Inc., Atlanta, GA), which estimates hemoglobin using nail bed images, reports LOA of ±4.43 g/dL for adults and ±3.54 g/dL for children [13]. In contrast, NiADA achieves a significantly narrower LOA range of +2.86 to -2.64 g/dL, indicating greater accuracy and precision. NiADA also outperforms the Astrim Fit monitoring device (Sysmex Corporation), which, when validated among school-age children, exhibited a mean bias of 0.17 ± 1.95 g/dL and LOA of -3.65 to +3.99 g/dL [14]. Furthermore, NiADA's performance is on par with devices such as Radical 7 (Masimo Corp., Irvine, CA) [15] (LOA = − 2.30, 1.72 g/dL) and EzeCHeck (EzeRx Health Tech Pvt. Ltd., Bhubaneswar, India) [16]. Notably, the latter devices require additional specialized and costly devices, whereas NiADA is a more accessible and cost-effective alternative. These results highlight NiADA's potential as a reliable and scalable tool for non-invasive hemoglobin estimation, particularly in resource-constrained settings.

One of the key strengths of the first study was the consecutive collection of venous and capillary blood samples, which minimized within-person variability and ensured robust comparisons. Furthermore, daily quality control procedures were implemented for the equipment, ensuring the reliability and consistency of the measurements. In the second study, the inclusion of observations and estimations from 15 medical professionals - most of whom hold advanced degrees in pathology - provided a comprehensive benchmark for evaluating NiADA's performance. For both of the studies, NiADA has shown consistency, though there are some variations because of different sample sizes. 

The study highlighted several limitations. NiADA was less effective at detecting severe anemia cases and showed reduced accuracy at the extreme ends of hemoglobin levels (>16 g/dL and <8 g/dL), likely due to imbalances in the training data. Addressing these issues may require the inclusion of additional real-world and synthetic data samples to enhance the algorithm’s training and performance. Furthermore, incorporating data from diverse geographic regions could make the NiADA model more robust.

Improving the quality of captured images is another key area for development. Discrepancies in the results may also have arisen from unknown medical conditions that impacted how blood hemoglobin levels were reflected in the eyelid’s color, highlighting the need for further investigation.

Future studies should focus on refining the software algorithm, improving the quality of image inputs, and identifying potential confounding factors to enhance NiADA’s reliability across a broader spectrum of hemoglobin levels.

## Conclusions

This study demonstrates that hemoglobin levels can be reliably estimated using images of the inner lower eyelid captured with standard smartphones. The NiADA application offers a transformative approach to community-based anemia diagnosis and monitoring. Its non-invasive, accessible, and cost-effective design makes it an ideal solution for resource-limited settings. NiADA has shown consistent performance across two different datasets, highlighting its robustness. Although NiADA is not intended to replace laboratory-based hemoglobin testing, it serves as an effective screening and monitoring tool. Hemoglobin is a critical biomarker of health, and NiADA’s real-time capabilities provide a scalable, affordable option for public health initiatives. By integrating NiADA into community health programs, public administrators and non-profit organizations can more effectively screen and monitor anemia prevalence, especially in vulnerable populations. This integration, especially when paired with nutritional interventions and supplement distribution, presents an ecologically sustainable and logistically practical alternative to traditional point-of-care devices. Beyond public health applications, NiADA also empowers individuals to manage their personal health, enabling at-home anemia screening and monitoring for preventive care and wellness. To maintain its reliability, the AI model underlying NiADA must be continually trained and updated with diverse, high-quality datasets, ensuring its adaptability to varying populations and conditions.

In summary, NiADA represents an innovative tool with significant potential to improve anemia management and public health outcomes globally. Its implementation can enhance population health monitoring and offer a sustainable, accessible solution to a widespread public health challenge.
